# Observations on the Growth and Irregularities of Children's Teeth

**Published:** 1845-09

**Authors:** 


### Bibliographical Notices.

***Observations on the Growth and Irregularities of Children's Teeth: followed by Remarks and Advice on the Teeth in general. To which is added a Short Essay on Artificial Teeth.*** By W. H. Mortimer, late Surgeon Dentist to the British Embassy at Paris. Second Edition, revised, pp. 129, 12mo. London, 1845.

As narrow as are the limits of this work, we have not had time to give it a careful perusal. We are not, therefore, prepared to speak of its merits nor point out its defects, so fully as we should be glad to do. The first edition, as we learn from the preface, was published during the author's residence in Paris; and in stating the object which he had in view in its publication, we cannot do better than quote his own language upon the subject. He says, it was "to instruct parents in an elementary knowledge of the growth of children's teeth." * * * "It was originally intended that this work should only treat of the growth and irregularities of children's teeth; but finding the subject would only allow of a short dissertation, without entering into long and unnecessary physiological digressions, it was thought advisable to preface it with a concise notice of the diseases and treatment during the painful process of dentition, and of adding a few remarks on the teeth in general."

The work certainly contains much good advice, and, as a whole, may be productive of some good. We should not, however, be led to believe, from the cursory reading which we have given it, that the author is a man of much experience, or that he had profited much from observation; and as the general reader is more liable to be led astray by the perusal of a work containing erroneous opinions and doctrines, than the professional man, it is important that books of this description should be as free from them as possible.

